# Evaluation of tobacco use and willingness to accept nicotine replacement therapy during stay in an acute inpatient psychiatric ward

**DOI:** 10.1192/bjo.2021.504

**Published:** 2021-06-18

**Authors:** Isabel Ganhao, Miguel Trigo, Afonso Paixao

**Affiliations:** Centro Hospitalar Psiquiatrico de Lisboa

## Abstract

**Aims:**

To evaluate tobacco smoking and willingness to try and to accept prescription of nicotine replacement therapy during psychiatric acute inpatient stay. When free to choose and use as desired (without imposing smoking cessation) patients may be open to nicotine replacement therapy.

**Background:**

Tobacco smoking interventions are increasingly in demand especially for difficult to treat patient populations, as are those with severe mental illness. Implementing totally smoke-free psychiatric inpatient units is challenging. Imposing smoking cessation and use of nicotine substitutes may or not be the best of strategies for smoking reduction and cessation in the mid and long term. Allowing the patient free choice as to trying and learning to use nicotine replacement therapy with supervision may constitute a more acceptable approach.

**Method:**

From 1/1/2020 to 28/1/2020 (four weeks), 40 of the 44 patients in a general adult psychiatric inpatient unit (4 patients were too sedated or too agitated to be evaluated), with smoking restricted to a designated area and only during the day, were briefly evaluated as to their tobacco smoking habits (cigarettes/day) and willingness to accept nicotine lozenges and patches and were invited to participate in a smoking cessation programme during or after discharge.

**Result:**

Of the 40 patients evaluated, 26 were male and 14 were female, average age was 34 years (age range from 19 to 79 years). Diagnostic hypotheses for patients at admission were: schizophrenic disorder/schizoaffective disorder 9, bipolar disorder 7, psychosis not otherwise specified 19, depressive disorder 1 and other 4. 20(50%) were current smokers and 20 (50%) were non or ex-smokers. The smokers reported smoking an average of 14 cigarettes/day. Only one patient refused to try nicotine lozenges and all other smokers accepted regular prescription of nicotine lozenges during inpatient stay. 5 patients (25% of smokers) asked for or accepted suggestion to try nicotine patches in combination therapy with nicotine lozenges with the goal of smoking cessation.

**Conclusion:**

Patients were open to a brief informal intervention targeting smoking behaviours and readily accepted trying nicotine lozenges and prescription during their inpatient stay. The regular use of nicotine replacement therapy by some patients encouraged other patients to try and accept therapy. In addition to the habitual tobacco sharing among patients, nicotine lozenges were also shared especially with newly admitted patients. The evaluation of the impact of this intervention will require a much longer period of time of implementation.

